# Improved preservation of ovarian tissue morphology that is compatible with antigen detection using a fixative mixture of formalin and acetic acid

**DOI:** 10.1093/humrep/deab075

**Published:** 2021-05-06

**Authors:** B V Adeniran, B D Bjarkadottir, R Appeltant, S Lane, S A Williams

**Affiliations:** 1 Nuffield Department of Women’s and Reproductive Health, Women’s Centre, John Radcliffe Hospital, University of Oxford, Oxford, UK; 2 Future Fertility Programme Oxford, Oxford, UK; 3 Department of Paediatric Oncology and Haematology, Children's Hospital Oxford, Oxford University Hospitals NHS Foundation Trust, Oxford, UK

**Keywords:** fixation, histology, immunohistochemistry, form-acetic, neutral buffered formalin, Bouin’s, ovary, human, mouse, sheep

## Abstract

**STUDY QUESTION:**

Can ovarian tissue morphology be better preserved whilst enabling histological molecular analyses following fixation with a novel fixative, neutral buffered formalin (NBF) with 5% acetic acid (referred to hereafter as Form-Acetic)?

**SUMMARY ANSWER:**

Fixation with Form-Acetic improved ovarian tissue histology compared to NBF in multiple species while still enabling histological molecular analyses.

**WHAT IS KNOWN ALREADY:**

NBF fixation results in tissue shrinkage in various tissue types including the ovary. Components of ovarian tissue, notably follicles, are particularly susceptible to NBF-induced morphological alterations and can lead to data misrepresentation. Bouin’s solution (which contains 5% acetic acid) better preserves tissue architecture compared to NBF but is limited for immunohistochemical analyses.

**STUDY DESIGN, SIZE, DURATION:**

A comparison of routinely used fixatives, NBF and Bouin’s, and a new fixative, Form-Acetic was carried out. Ovarian tissue was used from three different species: human (n = 5 patients), sheep (n = 3; 6 ovaries; 3 animals per condition) and mouse (n = 14 mice; 3 ovaries from 3 different animals per condition).

**PARTICIPANTS/MATERIALS, SETTING, METHODS:**

Ovarian tissue from humans (aged 13 weeks to 32 years), sheep (reproductively young i.e. 3–6 months) and mice (10 weeks old) were obtained and fixed in 2 ml NBF, Bouin’s or Form-Acetic for 4, 8, and 24 h at room temperature. Tissues were embedded and sectioned. Five-micron sections were stained with haemotoxylin and eosin (H&E) and the percentage of artefact (clear space as a result of shrinkage) between ovarian structures was calculated. Additional histological staining using Periodic acid-Schiff and Masson’s trichrome were performed on 8 and 24 h NBF, Bouin’s and Form-Acetic fixed samples to assess the compatibility of the new fixative with stains. On ovarian tissue fixed for both 8 and 24 h in NBF and Form-Acetic, immunohistochemistry (IHC) studies to detect FOXO3a, FoxL2, collagen IV, laminin and anti-Müllerian hormone (AMH) proteins were performed in addition to the terminal deoxynucleotidyl transferase nick end labelling (TUNEL) assay to determine the compatibility of Form-Acetic fixation with types of histological molecular analyses.

**MAIN RESULTS AND THE ROLE OF CHANCE:**

Fixation in Form-Acetic improved ovarian tissue morphology compared to NBF from all three species and either slightly improved or was comparable to Bouin’s for human, mouse and sheep tissues. Form-Acetic was compatible with H&E, Periodic acid-Schiff and Masson’s trichrome staining and all proteins (FOXO3a, FoxL2, collagen IV and laminin and AMH) could be detected via IHC. Furthermore, Form-Acetic, unlike NBF, enabled antigen recognition for most of the proteins tested without the need for antigen retrieval. Form-Acetic also enabled the detection of damaged DNA via the TUNEL assay using fluorescence.

**LARGE SCALE DATA:**

N/A

**LIMITATIONS, REASONS FOR CAUTION:**

In this study, IHC analysis was performed on a select number of protein types in ovarian tissue thus encouraging further studies to confirm the use of Form-Acetic in enabling the detection of a wider range of protein forms in addition to other tissue types.

**WIDER IMPLICATIONS OF THE FINDINGS:**

The simplicity in preparation of Form-Acetic and its superior preservative properties whilst enabling forms of histological molecular analyses make it a highly valuable tool for studying ovarian tissue. We, therefore, recommend that Form-Acetic replaces currently used fixatives and encourage others to introduce it into their research workflow.

**STUDY FUNDING/COMPETING INTEREST(S):**

This work was supported by the Oxford Medical Research Council Doctoral Training Programme (Oxford MRC-DTP) grant awarded to B.D.B. (Grant no. MR/N013468/1), the Fondation Hoffmann supporting R.A. and the Petroleum Technology Development Fund (PTDF) awarded to B.V.A.

## Introduction

Fixation is the cornerstone of histopathology as it enables tissue preservation in an archival form thereby enabling the long-term study of cellular architecture and tissue composition. A number of different fixatives are commercially available, with the aldehyde group of fixatives serving as the most common for tissue fixation. Formaldehyde, which belongs to the aldehyde group, is the most widely used fixative and acts by forming covalent chemical bonds (commonly referred to as cross-links) between and within certain regions of protein structures thereby preserving the tissue (Fraenkel-Conrat and Olcott, 1948; Gustavson. 1956). It is commonly supplied as 10% (v/v) neutral buffered formalin (NBF) and comprises approximately 4% formaldehyde in PBS. Twenty-four hours fixation in NBF is the accepted standard for pathologists for most tissue types although fixation times may vary depending on tissue size in research use (Howat and Wilson, 2014).

The pitfall to using NBF is tissue-type-dependent shrinkage (Siu *et al.*, 1986; Pritt *et al.*, 2005; Jonmarker *et al.*, 2006; Chen *et al.*, 2012). Shrinkage has been reported in many tissue types including the prostate (Jonmarker *et al.*, 2006), oesophagus (Siu *et al.*, 1986), head and neck tissue (Chen *et al.*, 2012). NBF-induced shrinkage has regularly been observed in ovarian tissue but rarely acknowledged as it is considered an unavoidable issue. Marked shrinkage can adversely result in a misrepresentation of data for analytical purposes. In cancer studies, NBF-induced shrinkage resulted in decreased tumour size measurements and a misdiagnosis by underestimating the stage of the tumour (Hsu *et al.*, 2007; Tran *et al.*, 2015). In our laboratory, we have observed shrinkage in NBF fixed ovarian tissue, with follicles being particularly susceptible, and observed that NBF-induced shrinkage could affect ovarian tissue analysis (unpublished data).

The ovary is a dynamic organ with a high level of heterogeneity in terms of cell types, follicle stages, and structure. Morphological analyses of ovarian tissue before and after experimental manipulation provide an insight into follicle development and, hence, ovarian function and these rely heavily on observations of ovarian tissue histology following fixation. Therefore, accurate histological evaluation of ovarian tissue is critical. Ovarian tissue analysis involves classifying follicles according to their developmental stage in addition to assessing follicle health on fixed sections, both of which are employed by many research groups studying ovarian function (Fenwick and Hurst, 2002; Pangas *et al.*, 2007; Chambers *et al.*, 2010; Fenwick *et al.*, 2013; Kim *et al.*, 2015; Fabbri *et al.*, 2016; Stefansdottir *et al.*, 2016; Chiti *et al.*, 2017; McLaughlin *et al.*, 2018; Lee *et al.*, 2019; Winship *et al.*, 2019).

Shrinkage induced by NBF affects ovarian tissue architecture at the level of individual cells. It can be morphologically characterised as shrunken ooplasm within oocytes, condensed nuclei, shrunken stromal, and granulosa cells with ‘clear space’ seen in between the various cell types. NBF-induced morphological alterations also form part of the criteria involved in assessing follicle health, such as assessing whether there is contact between the oocyte and granulosa cells (Pampanini *et al.*, 2019; Walker *et al.*, 2019). This results in a dilemma wherein follicle death may be histologically presumed where, in fact, the fixative is the primary cause of the morphological appearance. Downstream molecular assays of cell death may be utilised to affirm or disprove observations, but this can be time-consuming and expensive when not required.

NBF is widely used in ovarian tissue fixation and is considered superior to other commercially available fixatives because it enables both routine histology and the detection of numerous protein molecules using immuno-labelling (Howat and Wilson, 2014). However, as outlined above, NBF can cause tissue shrinkage, particularly in ovarian tissue and, therefore, alternative solutions have been sought. Bouin’s solution (formaldehyde, picric acid, and acetic acid in an aqueous solution) has been demonstrated to preserve ovarian tissue morphology better than NBF and is, therefore, often used for the fixation of ovarian tissue for histological analyses. Its use is, however, limited to histology owing to its protein coagulative properties which make it poorly suited to immuno-labelling (Howat and Wilson, 2014). Thus, the search for the perfect fixative carries on.

The optimal fixative should allow for good morphological detailing, unbiased diagnosis of disease and evaluation of developmental stages amongst other histological criteria. The fixative should also allow for most if not all protein types to be easily recognisable/identified on fixed tissue, as is the case for NBF. In addition to these, the fixative should enable the preservation of DNA/RNA to enable sequencing/detection. Our aim was, therefore, to develop a fixative that was capable of preserving tissue as the ‘ideal’ fixative outlined above, focussing on the most commonly used applications for the study of ovarian tissue, namely histological and immunohistochemical (IHC) staining.

In this study, we compare a new fixative comprising NBF with 5% acetic acid (termed Form-Acetic) with two routinely used fixatives, NBF and Bouin’s solution, by analysis of tissue morphology and antigen availability for IHC in ovarian tissue from human, sheep, and mouse. We tested 5% acetic acid in NBF since this is the concentration present in Bouin’s solution. We demonstrate that fixation with Form-Acetic resulted in improved morphological preservation in addition to also supporting downstream assays, such as IHC and terminal deoxynucleotidyl transferase nick end labelling (TUNEL), in ovarian tissue from multiple species.

## Materials and methods

### Ethics and tissue collection

#### Human

The use of human tissue was approved by the Health Research Authority South Central—Oxford B Research Ethics Committee (REC reference 14/SC/0041). Fresh whole ovaries (n = 3 patients) and frozen ovarian cortical tissue (n = 2 patients) were obtained from the Oxford Cell and Tissue Biobank (OCTB) (Table I); OCTB obtained consent from the patients to donate this tissue for research. None of the patients had previously received chemotherapy or radiotherapy. The ovaries/ovarian tissue were removed as part of surgical procedures and donated for research.

**Table I:** Characteristics of human samples and assays performed.

Patient		1	2	3	4	5
Age		<1 year	32 years	29 years	9 years	13 years
Fresh/frozen		Fresh	Fresh	Fresh	Frozen	Frozen
Assays	H&E		Y	Y		Y
	PAS		Y	Y		
	Trichrome		Y	Y		
	FOXO3a	Y	Y	Y	Y	Y
	FoxL2		Y	Y	Y	Y
	Laminin	Y	Y		Y	
	Collagen IV	Y	Y		Y	
	AMH (DAB)	Y	Y	Y	Y	Y
	AMH (IF)	Y	Y		Y	
	TUNEL	Y			Y	

Y = analysis performed, yr = years of age, DAB = 3’-diaminobenzidine, IF = immunofluorescence, greyed out = not tested H&E: haemotoxylin and eosin, PAS: Periodic acid-Schiff, FOXO3a: fork-head box O3 (transcription factor), FoxL2: fork-head box protein L2 (transcription factor), anti-Müllerian hormone (AMH; hormone), laminin α1 and collagen IV (extracellular matrix proteins).

The fresh ovaries were transported and dissected in cold (4°C) Leibovitz L-15 media (Sigma, Gillingham, UK, L5520) to isolate the ovarian cortex. The frozen cortical tissue was thawed in solutions of decreasing concentrations of ethylene glycol (1.0, 0.5, and 0 M) (Sigma, 324558), 0.1 M sucrose (Sigma, S7903), and 3 mg/mL human serum albumin (HSA; Sigma, A1653) for 5 min each at room temperature, using a rocking motion. All cortical tissue was further dissected in fresh L-15 into pieces approximately ≤1 mm^3^. Processing time between tissue collection from the OCTB and fixation was approximately 1 h.

#### Sheep

Female reproductive tracts were obtained shortly after death at a local abattoir. Tracts were assessed visually and based on the size of the ovaries and uterus, appeared to be from young animals (3-6 months). Pairs of visually normal sheep ovaries (n = 3) were collected and transported in L-15 (Gibco, Loughborough, UK, 11415049) supplemented with 2.5 µg/mL Amphotericin B (Gibco, 15290-018), 100 IU/mL penicillin and 100 mg/mL streptomycin (Sigma, P0781) on ice to the laboratory (approximately 1 h). In fresh L-15, the ovarian cortex was isolated from pairs of ovaries and dissected into pieces approximately ≤1 mm^3^. Processing time between dissection and fixation was approximately 1 h.

#### Mouse

Pairs of ovaries were obtained from euthanised wild-type C57BL/10 mice (27 ovaries from 14 mice) at 10 weeks of age. Each ovary was dissected out and rinsed in Dulbecco’s PBS (DPBS, Sigma, D8662) prior to fixation. Time between ovary collection and fixation was approximately 1 h.

### Tissue allocation, fixation, embedding and sectioning

Samples from each species were fixed by immersion in 2 ml of 10% NBF (VWR, Poole, UK, 11699408), Bouin’s (Sigma, HT10132) and Form-Acetic (5% acetic acid in NBF) (acetic acid, Merck, Feltham, UK, 1.00063.1011) solution for 4, 8, and 24 h at room temperature with gentle rocking. The volume of fixative was at least 10× the volume of the sample. For human and sheep fixation, three pieces of ovarian cortical tissue from each individual were fixed in each condition. Samples from individual patients and each sheep were randomly allocated to each fixative condition at the same time and could be traced to particular individuals. For mice, ovaries were fixed whole with one ovary from three different mice in each condition. Post fixation, the ovarian tissue/ovaries were washed in 70% ethanol twice for 5 min and stored in 70% ethanol for a minimum of 1 h and a maximum of 6 months before embedding; all samples fixed at the same time (i.e. in all conditions) were embedded at the same time to control for duration in 70% ethanol. For embedding, fixed ovarian samples were dehydrated in increasing concentrations of ethanol (70%, 80%, 95%, 100% ×3), cleared in xylene (×3) and embedded in paraffin wax. All samples were serially sectioned at 5 µm.

### Histological staining

Sections from the fixed human, sheep, and mouse specimen were stained with haemotoxylin and eosin (H&E; Haemotoxylin, Gill no 2, Sigma, GHS232; Eosin Y, Sigma, HT110332), Periodic acid-Schiff (PAS; Periodic acid solution, Sigma, 3951; Schiff’s reagent, Merck, 1.09033.0500) and Masson’s trichrome (Abcam, Cambridge, UK, ab150686) stain. In brief, 5 µm sections were dewaxed (×3) in xylene and rehydrated in decreasing concentrations of ethanol (×3 100%, 90%, 70%, 50%) before proceeding with further staining. For H&E, sections were incubated in haemotoxylin for 2 min, de-stained (1% hydrochloric acid in 70% ethanol) for 5 s, washed in 80% ethanol for 1 min, followed by a brief incubation (1 s) in eosin before dehydrating in ethanol (95% and 100% ×3) and clearing in xylene (×3). For PAS, sections were incubated in periodic acid for 5 min, Schiff’s reagent for 15 min and haemotoxylin for 90 s before dehydrating in ethanol (95% and 100% ×3) and clearing in xylene (×3). For Masson’s trichrome, sections were incubated in pre-heated (60°C) Bouin’s solution for 1 h, followed by Weigert’s iron haematoxylin for 5 min, Biebrich scarlet/acid fuchsin solution for 15 min, phosphomolybdic/phosphotungstic acid solution for 14 min, aniline blue for 9 min and acetic acid for 5 min before dehydrating in ethanol (95% ×2 and 100% ×2) and clearing in xylene (×2). Stained slides were mounted using DPX mountant (Sigma, 06522), examined under a light microscope (Leica DM2500) and images captured using the QImaging Micropublisher 6 camera and accompanying Ocular imaging software (QImaging, Surrey, Canada).

### Histological artefact assessment

Three histological assessments were performed on H&E-stained sections to determine the morphological integrity of ovarian tissue after fixation and these involved assessing follicle integrity (the amount of clear space, as a result of cellular shrinkage due to fixation, observed within the follicle), follicle-stroma integrity (space between the follicle and the surrounding stroma) and stroma integrity (space between stromal cells). Follicles at the primordial to secondary stage were assessed for the follicle and follicle-stroma integrity categories, and regions considered as artefact (clear space) for each category were measured using ImageJ (National Institutes of Health, Bethesda, MD, USA) (Supplementary Fig. S1). In assessing follicle integrity, regions of artefact were identified and the total area of these measurements within the follicle was calculated as a percentage of the total follicular area. Only follicles with a visible oocyte nucleus or nuclear membrane were included in this assessment. Follicle-stroma integrity was determined by measuring the total perimeter of non-interaction between the follicle and the stroma and calculated as a percentage of the total follicular perimeter. Stroma integrity was determined by measuring the total area of artefact within the stroma as a percentage of the total stroma area for each section analysed, using thresholding. Thresholding involved the conversion of images to 8 bits, which changed the coloured image to black and white; carried out for all sections. Threshold values were adjusted using the original colour image as a reference to discriminate artefact from stained regions of tissue. Spaces due to blood vessels and previously assessed categories (follicle and follicle-stroma integrity) were excluded from the stroma integrity analysis. To avoid double counting of follicles, analysed sections of human and sheep ovaries were at least 25 µm apart while mouse ovary sections were at least 100 µm apart. Sections selected for analysis were distributed throughout the tissue. All sections were assessed blindly.

### Ovarian follicle classification

Follicles were classified according to established criteria (Pedersen and Peters, 1968; Gougeon, 1996; Lundy *et al.*, 1999; Grasa *et al.*, 2015; Walker *et al.*, 2019); primordial (oocyte is surrounded by a single layer of flattened pre-granulosa cells), transitional (a mixed layer of flattened and cuboidal granulosa cells surrounding the oocyte), primary (minimum of a complete layer of cuboidal cells surrounding the oocyte), and secondary (two layers of cuboidal granulosa cells surrounding the oocyte). Mouse pre-antral follicles were defined as having many granulosa cell layers with interspersed fluid filled areas and antral follicles were those that contained many layers of granulosa cells and a large antral cavity (Pedersen and Peters, 1968).

### Immunohistochemistry using diaminobenzidine and immunofluorescence

All IHC/immunofluorescence (IF) experiments were performed at least twice for each individual sample tested. Sections containing follicles of appropriate developmental stage were selected for IHC/IF, but not all patient samples were used for IHC/IF analyses owing to the limited numbers of follicles on sections (Table I).

Immunohistochemical evaluation was performed on sections fixed in NBF and Form-Acetic for 8 h and 24 h for the following antibody targets: fork-head box O3 (FOXO3a; transcription factor), fork-head box protein L2 (FoxL2; transcription factor), anti-Müllerian hormone (AMH; hormone), laminin α1 and collagen IV (extracellular matrix proteins). Following dewaxing and rehydration, sections were subject to no antigen retrieval (No AR) or heat-induced antigen retrieval (AR) by microwave heating for 10 min with a further 20 min cool-down period using either sodium citrate (pH 6.0) (for FOXO3a and collagen IV) or 1× antigen unmasking solution, Tris-based (Vector Laboratories, Peterborough, UK, H-3301) (for FoxL2, laminin and AMH). Sections (for DAB staining only) were then treated with 3% hydrogen peroxide for 5 min and washed in PBS (20 mM phosphate, 150 mM NaCl, pH 7.4) for 5 min two times to block endogenous peroxidase activity.

To detect AMH, FOXO3a, collagen IV and laminin proteins, sections were blocked in 5% normal goat serum (NGS; Vector) in PBS with 0.05% Tween 20 (Fisher Scientific, Loughborough, UK) (PBS-T) to prevent non-specific binding for 1 h at room temperature. Sections were incubated in 5% NGS in PBS-T overnight at 4°C with mouse monoclonal anti-AMH (1:100; Biorad, Dalkeith, UK, MCA2246), rabbit monoclonal anti-FOXO3a (1:100; Cell Signaling Technology, UK, 12829S), rabbit polyclonal anti-collagen IV (1:100; Millipore, Hertfordshire, UK, AB8201), or rabbit polyclonal anti-laminin α1 (1:30; Sigma, L9393). For FoxL2 detection, sections were blocked with 5% rabbit serum (Sigma, R9133) in PBS-T for 1 h at room temperature followed by goat polyclonal anti-FoxL2 (1:500; Novus Biologicals, Oxon, UK, NB100-127755) overnight at 4°C. The sections were washed three times for 5 min in PBS-T and incubated in the following secondary antibodies: biotinylated goat anti-mouse IgG (Vector Laboratories, UK, BA-9200, 1:100 dilution) for AMH, goat anti-rabbit IgG (Vector Laboratories, BA-1000, 1:200 dilution) for FOXO3a, collagen IV and laminin, and rabbit anti-goat (Vector Laboratories, BA-5000, 1:300 dilution) for FoxL2 for 1 h at room temperature. Negative control sections were treated with the following appropriate IgG antibodies: purified mouse IgG κ isotype (Biolegend, London, UK, 401401), rabbit mAb IgG XP isotype control (Cell Signaling Technology, DA1E) and for FoxL2, the negative control section had the primary antibody omitted. Following secondary antibody incubation, sections were washed in PBS-T for 3 min, three times.

Staining was achieved using the Vectastain ABC Elite kit (Vector Laboratories) for 30 min at room temperature followed by a final development with a DAB peroxidase substrate kit (Vector Laboratories). Slides were counterstained in Gills 2 haemotoxylin, mounted and images were captured using the QImaging Micropublisher 6 camera and accompanying Ocular imaging software (QImaging, Canada) and the Lumenera Infinity 5 camera and accompanying Infinity Analyze software (Teledyne Lumenera, Nepean, Canada).

A portion of slides labelled to detect AMH (after AR) were visualised using fluorescence. Following secondary antibody incubation as described above, sections were incubated with streptavidin Alexa Fluor 568 conjugate (1:200; Thermo Fisher, S11226) for 30 min at room temperature. Slides were washed in PBS-T and counterstained with 5 μg/mL DAPI (Sigma, D9542) before being mounted with Vectashield^®^ Hardset^TM^ Antifade mounting medium (Vector Laboratories, H-1400).

Fluorescent-labelled sections were imaged under a fluorescent microscope (Leica DMRBE) with LED illumination and DAPI-FITC-TRITC filters. Images were captured using the QImaging Retiga R3 camera and Velocity software. All sections were imaged using the same acquisition settings (laser power, gain, and exposure). Post-imaging processing was performed using ImageJ to enhance qualitative aspects (brightness and contrast) of the red channel (fluorescent AMH labelling) to allow for increased visibility of figures in print. All adjustments were made following best-practice guidelines (Lee and Kitaoka, 2018), with all images (control and experimental across all groups) treated and adjusted identically with adjustments applied uniformly to whole images. Imaging processing steps involved adjusting the upper and lower limits of the display range by modifying the minimum and maximum settings of the image (16-bit). The optimal range was first determined using the sample with the lowest level of observed signal (24 h Form-Acetic) and the corresponding IgG negative control to ensure high signal visibility with minimal background. The same settings were then applied to all other sample groups. The blue channel (DAPI counterstain) was not altered.

### TUNEL assay

Detection of double-stranded DNA breaks was performed using the Click-IT™ Plus TUNEL assay (Invitrogen, UK, C10618) according to the manufacturers’ instructions on 8 h and 24 h NBF and Form-Acetic fixed human and mouse sections. Slides were counterstained with 5 μg/mL DAPI, mounted with Vectashield Hardset Antifade mounting medium and imaged under a fluorescent microscope with LED illumination and DAPI-FITC-TRITC filters. Positive control sections were treated with 1 IU/ml DNase I (Invitrogen, 18047019).

### Statistical analyses

All statistical analyses were performed using R statistical software version 4.0.2. (R Foundation for Statistical Computing, Vienna, Austria). Linear mixed effects regressions (lm4e package; Bates *et al.* 2015) were used to detect the effect of fixative and duration on histological scores (follicle integrity, follicle-stroma integrity, and stroma integrity), where individual was included as a random effect to adjust for individual variation. Data are presented as mean ± SEM and statistical significance was defined as *P* < 0.05.

## Results

### Form-Acetic preserves and improves ovarian tissue morphology

**Figure 1.**
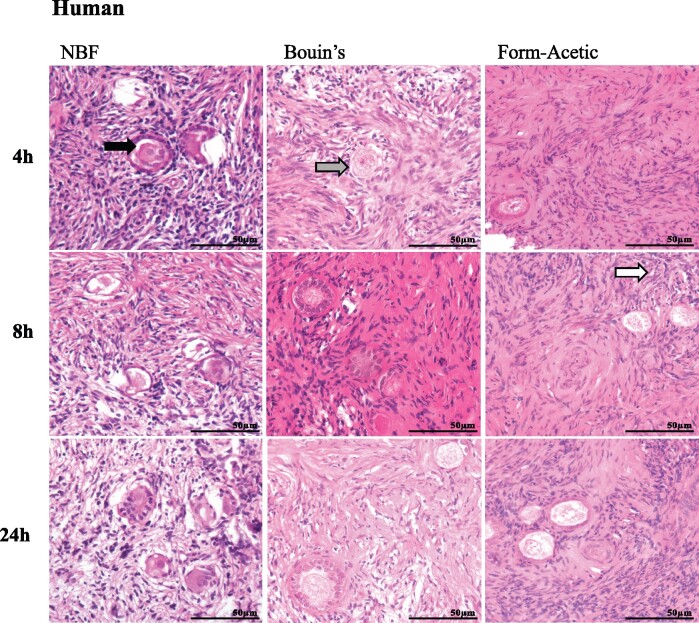
**Representative images of haemotoxylin & eosin stained human ovarian sections prepared with different fixatives**. Prior to haemotoxylin & eosin (H&E) staining, human ovarian tissue sections were fixed in neutral buffered formalin (NBF), Bouin’s and Form-Acetic (NBF with 5% acetic acid) solution for 4 h, 8 h, and 24 h. Arrows highlight regions of artefact including: intact follicles receding away from the stroma (follicle-stroma integrity; grey arrow), shrunken ooplasms within oocytes (follicle integrity; black arrow), granulosa cell nuclei condensation, and space within the stroma (stroma integrity; white arrow). Images are representative of human ovarian cortex samples, n=3 patients per condition (fresh n=2, frozen n=1).

**Figure 2.**
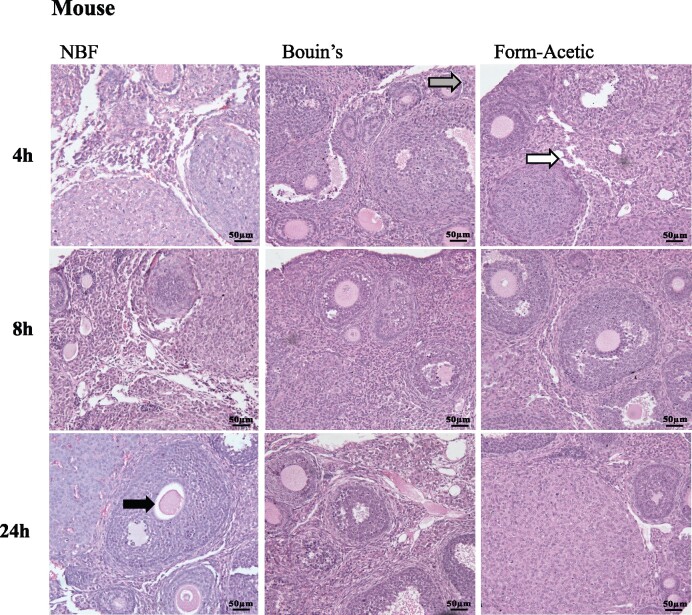
**Representative images of H&E stained mouse ovarian sections prepared with different fixatives.** Prior to H&E staining, mouse ovarian tissue sections were fixed in NBF, Bouin’s and Form-Acetic (NBF with 5% acetic acid) solution for 4 h, 8 h, and 24 h. Arrows highlight regions of artefact including: intact follicles receding away from the stroma (follicle-stroma integrity; grey arrow), shrunken ooplasms within oocytes (follicle integrity; black arrow), granulosa cell nuclei condensation and space within the stroma (stroma integrity; white arrow). Images are representative of mouse ovaries, n=3 animals per condition.

**Figure 3.**
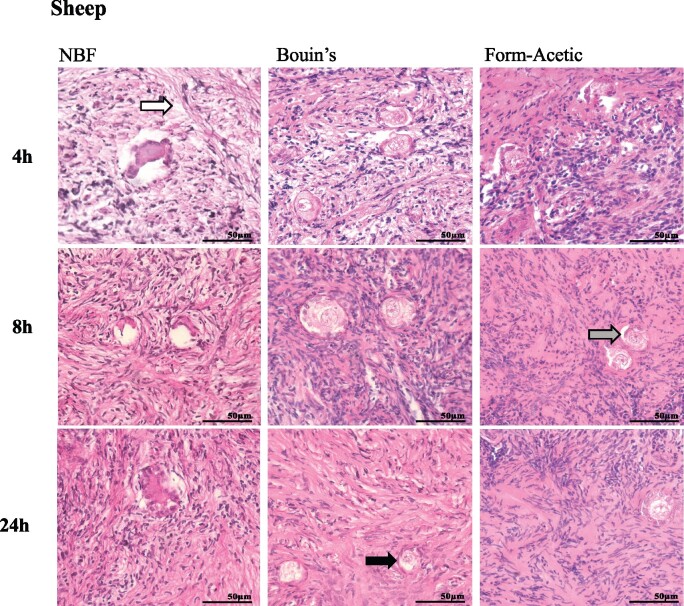
**Representative images of H&E stained sheep ovarian sections prepared with different fixatives.** Prior to H&E staining sheep ovarian tissue sections were fixed in NBF, Bouin’s and Form-Acetic (NBF with 5% acetic acid) solution for 4 h, 8 h, and 24 h. Arrows highlight regions of artefact including: intact follicles receding away from the stroma (follicle-stroma integrity; grey arrow), shrunken ooplasms within oocytes (follicle integrity; black arrow), granulosa cell nuclei condensation, and space within the stroma (stroma integrity; white arrow). Images are representative of sheep ovarian cortex samples, n=3 animals per condition.

Ovarian tissue from human, sheep, and mouse were fixed in NBF, Bouin’s, and Form-Acetic for 4, 8, and 24 h (Figs 1, 2, and 3). The fixed tissue was processed and stained with H&E. Within the stroma of NBF fixed tissue, artefact was often seen. NBF also introduced artefact that affected follicle-stroma integrity wherein intact follicles receded away from the stroma. NBF also caused shrunken ooplasms within oocytes and granulosa cell nuclei condensation, thereby affecting the follicle integrity (Figs 1, 2, and 3). In Bouin’s fixation, all three categories (follicle integrity, follicle-stroma integrity, stroma integrity) were affected in human and sheep tissues while in mouse, the stroma integrity was mainly affected. Infrequently seen in Form-Acetic fixed sections was artefact that affected all three categories (Figs 1, 2, and 3).

**Figure 4.**
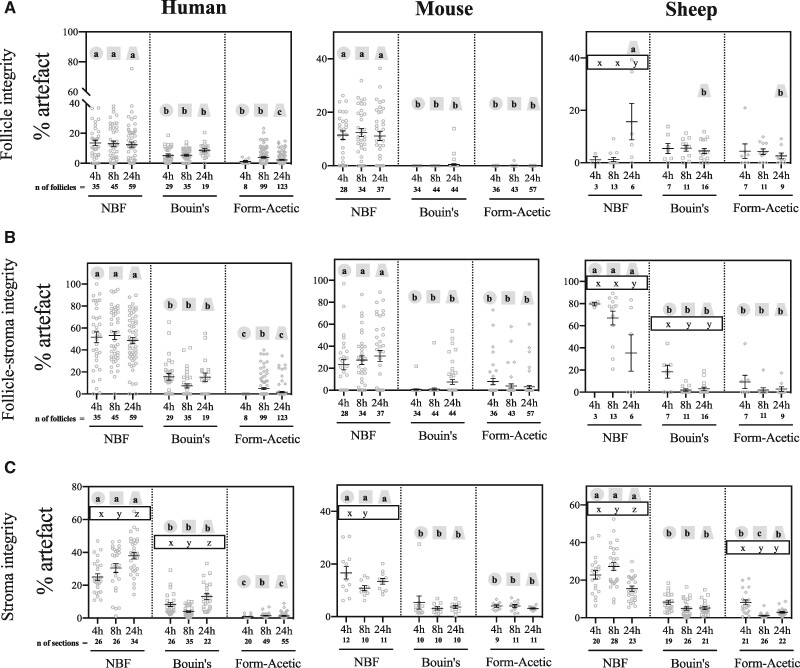
**Histological assessments of fixative conditions using H&E stained human, mouse and sheep ovarian sections.** Ovarian sections of human, mouse and sheep were fixed in NBF, Bouin’s and Form-Acetic (NBF with 5% acetic acid) for 4 h, 8 h, and 24 h. Histological assessments were performed to determine the morphological integrity of follicles, the follicle and stroma interaction, and the stroma. The percentage of ‘clear space’ (referred to as artefact) was measured to determine follicle integrity (A, top panel), follicle-stroma integrity (B, middle panel) and stroma integrity (C, bottom panel) after fixation in different fixatives. Percentages are represented as grey symbols with the mean ± SEM (in black) also shown. Calculations were performed to determine the degree of artefact associated with each condition. Significance between any two variables was determined with linear mixed effects regressions (lm4e package; Bates *et al.* 2015) using R statistical software version 4.0.2. A significant difference between variables is indicated by different letters on the graph (*P*<0.05). The letters a/b/c are used to denote significance between the different fixatives at a specific time-point. Each time-point, independent of the fixative, has the same shape around letters of significance to indicate that comparisons are between the fixative groups at that particular time. In contrast, the letters *x*/*y*/*z* denote significance within a fixative group only. The letters are within the same shape outline to show that comparisons are within the fixative group between the different times. Where no letter is seen to represent significance between or within groups, this suggests that no significant difference was seen between conditions. Further information on significance levels is provided in Supplementary Table SI. Images used for analysis were from n=3 different individuals per condition.

The percentage of tissue shrinkage after fixation was measured using ImageJ. Table I provides details on patient samples involved in the histological analyses. Following histological assessments, fixation in Form-Acetic or Bouin’s resulted in lower levels of artefact in human and mouse ovarian samples compared to NBF for all three categorical measures at all three-time points: 4, 8, and 24 h. For sheep ovarian samples, the level of artefact was reduced at all three time points for two of the three categories (follicle-stroma integrity and stroma integrity) whereas follicle integrity was improved by Form-Acetic compared to NBF at 24 h, but not 4 or 8 h (Fig. 4).

When comparing Form-Acetic to Bouin’s for mouse ovarian sections, the level of artefact in sections was equivalent for all three artefact categories for the same duration of fixation. When comparing Bouin’s and Form-Acetic for both the human and sheep samples, where there was a significant difference between the fixatives the level of artefact in Form-Acetic was always lower (Fig. 4).

When comparing the duration of fixation (4, 8, and 24 h) within a category and a species, the level of artefact in NBF fixed tissues differed significantly in five of the nine comparisons (Fig. 4) as compared to two of nine for Bouin’s and one of nine for Form-Acetic. Moreover, for some measures, integrity improved with duration in NBF (human stroma and sheep follicle integrity), but for others, integrity decreased with time (sheep follicle-stroma) or increased from 4 to 8 h then decreased at 24 h (stroma integrity). This indicated that NBF-induced artefact is less predictable and more variable with duration of fixation than Bouin’s and Form-Acetic.

In ovine samples, 4 h fixation in Form-Acetic was more comparable to Bouin’s fixed samples (Fig. 4) and resulted in increased artefact compared to 8 and 24 h fixation. Based on these data, we focussed on 8 and 24 h for further analyses.

The above describes comparisons made between the different fixatives for each time point, and for each fixative solution for different fixation durations. Further comparisons were performed between the groups and the results can be found in Supplementary Tables SI, SII, and SIII.

### Form-Acetic fixation is compatible with histological staining

To determine the compatibility of Form-Acetic with other histological stains, PAS and Masson’s trichrome stains were performed on 8 and 24 h NBF, Bouin’s and Form-Acetic fixed sections.

#### Periodic acid-Schiff

**Figure 5.**
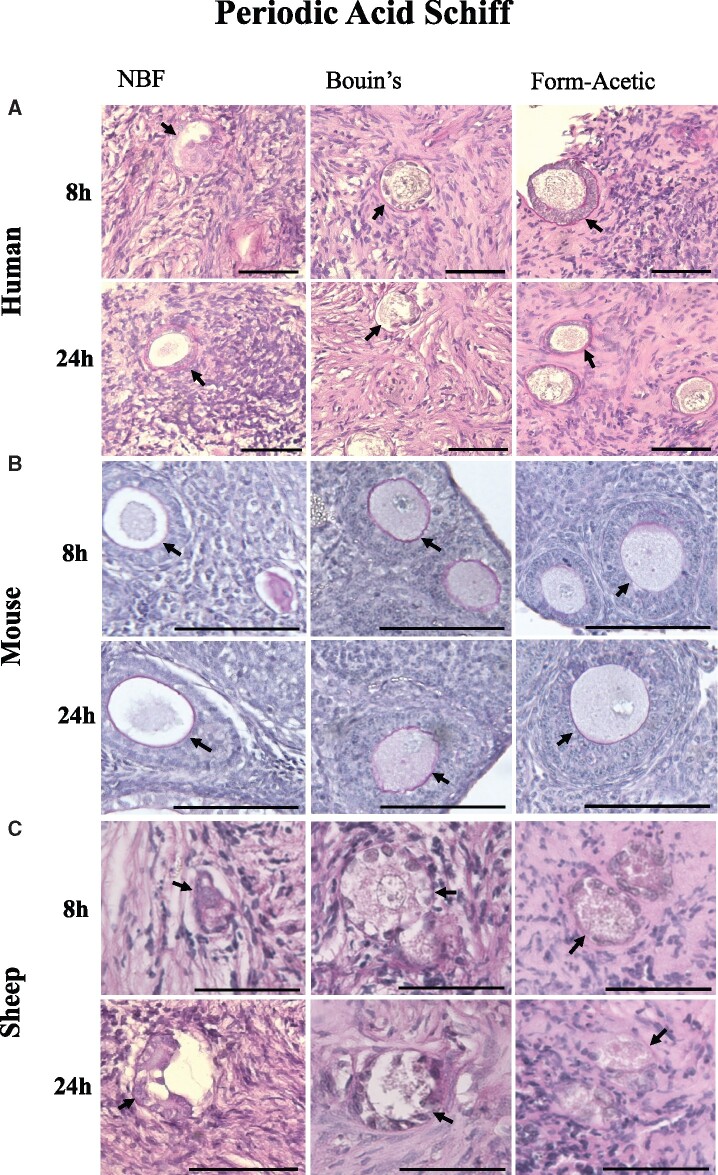
**Periodic acid Schiff stain of human, mouse and sheep ovarian tissue prepared with different fixatives**. Periodic acid Schiff (PAS) staining was performed on (A) human, (B) mouse and (C) sheep ovarian sections fixed in NBF, Bouin’s, and Form-Acetic (NBF with 5% acetic acid) for 8 h and 24 h. Images are representative of human ovarian cortex samples, n=2 patients per condition (fresh n=2), sheep ovarian cortex samples, n=3 animals per condition, and mouse ovaries, n=3 animals per condition. Scale bar represents 50 µm.

In human ovarian tissue, carbohydrate moieties in the basement membrane and zona pellucida of follicles were distinctly stained magenta by the PAS (Fig. 5A) irrespective of which fixative was used. In mouse sections, the zona pellucida was distinctly magenta in all conditions (Fig. 5B). In sheep sections, the basement membrane was also faintly magenta in all conditions but much less compared to the mouse and human samples (Fig. 5C).

##### Masson’s trichrome

**Figure 6.**
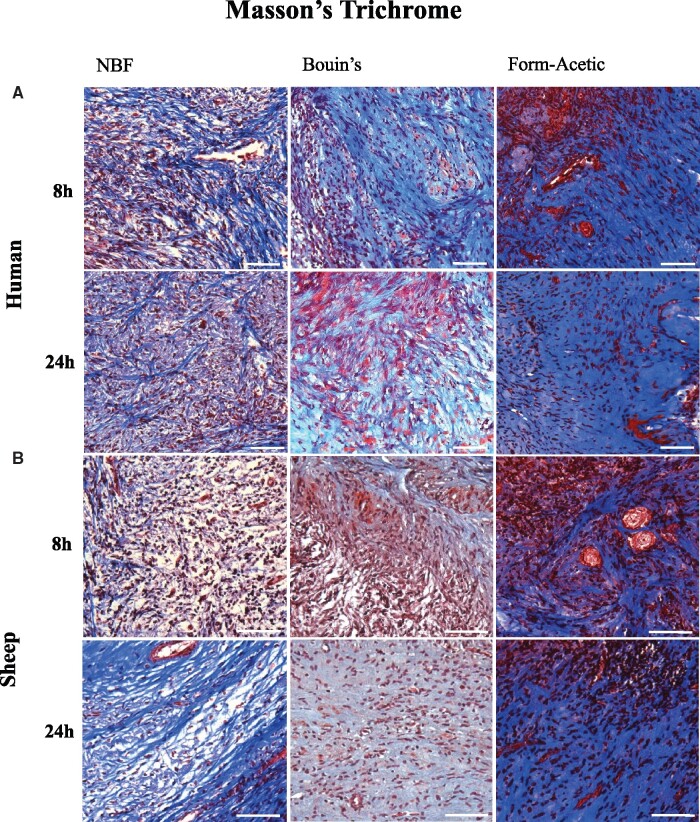
**Masson’s trichrome stain of human and sheep ovarian tissue prepared with different fixatives.** Masson’s trichrome staining was performed on (A) human and (B) sheep ovarian sections fixed in NBF, Bouin’s, and Form-Acetic (NBF with 5% acetic acid) for 8 h and 24 h. Images are representative of human ovarian cortex samples, n=2 patients per condition (fresh n=2) and sheep ovarian cortex samples, n=3 animals per condition. Scale bar represents 50µm.

Masson’s trichrome is an extracellular matrix dye, which stains collagen blue, nuclei blue-black, and muscles/cytoplasm red. Staining was performed on human and sheep samples only as mouse ovarian tissue contains very little collagen-enriched stroma (Berkholtz *et al.*, 2006). All fixatives were compatible with the Masson’s trichome stain, revealing a similar staining pattern of the various cellular structures between conditions (Fig. 6A and B). The stain also revealed the clear spaces introduced by the fixation of ovarian tissues.

### Form-Acetic is compatible with immunohistochemical studies

To determine whether Form-Acetic was compatible with immuno-labelling, NBF and Form-Acetic fixed human, mouse, and sheep samples (8 and 24 h) were subject to IHC, with and without AR. In human and mouse, a range of antibodies, encompassing different antigen categories, were tested: FOXO3a, FoxL2, collagen IV, laminin, and AMH, using DAB for visualisation. Compatibility of Form-Acetic with fluorescent detection was also validated in human tissue using AMH labelling with AR. Labelling for sheep samples was carried out for AMH, collagen IV, and laminin proteins only owing to poor antigenicity of FoxL2 and FOXO3a antibodies to sheep antigens. Table I provides details on patient samples involved in IHC analyses. Experiments using NBF and Form-Acetic fixed sections for each species were carried out in the same assay (for each antigen) and subjected to the same DAB exposure time; DAB exposure times varied depending on antibody used.

### Form-Acetic allows detection of a nuclear/cytoplasmic transcription suppressor—FOXO3a

**Figure 7.**
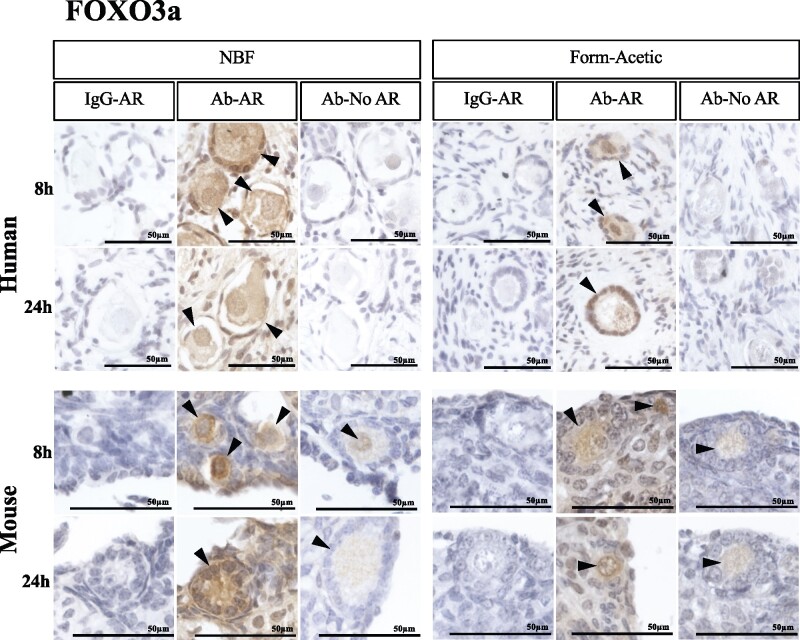
**Detection of FOXO3a using immunohistochemistry in human and mouse ovarian tissue preserved with different fixatives.** Following the fixation of ovarian tissue in NBF and Form-Acetic (NBF with 5% acetic acid) solutions for 8 h and 24 h, immunohistochemistry (IHC) was performed on human and mouse ovarian sections. Fork-head box O3 (FOXO3a) (a transcription factor), was detected in the oocytes of primordial to primary follicles. Follicles/cells positively stained are indicated by the arrowheads. IgG-AR: isotype IgG control was applied on the section treated with antigen retrieval (AR), Ab-AR: antibody was applied, and an AR step was performed, Ab-No AR: antibody was applied, and no AR step was performed. Images are representative of experiments using human ovarian cortex samples, n=5 patients per condition (fresh n=3, frozen n=2), and mouse ovaries, n=3 animals per condition. IHC experiments were performed at least twice for each sample. NB: The 12 images in each panel for a single species were all from the same experiment with the same DAB exposure enabling direct comparison between the samples.

FOXO3a was detected in the oocytes and granulosa cells of primordial and primary follicles of AR-treated human and murine sections fixed for 8 and 24 h in NBF and Form-Acetic (Fig. 7). However, when AR was not performed, FOXO3a was not detected in human ovarian sections irrespective of fixation (Fig. 7). In mouse samples without AR, however, FOXO3a was detected faintly in both Form-Acetic and NBF fixed sections at both time points (Fig. 7).

### Form-Acetic allows nuclear detection of a transcription factor—FoxL2

**Figure 8.**
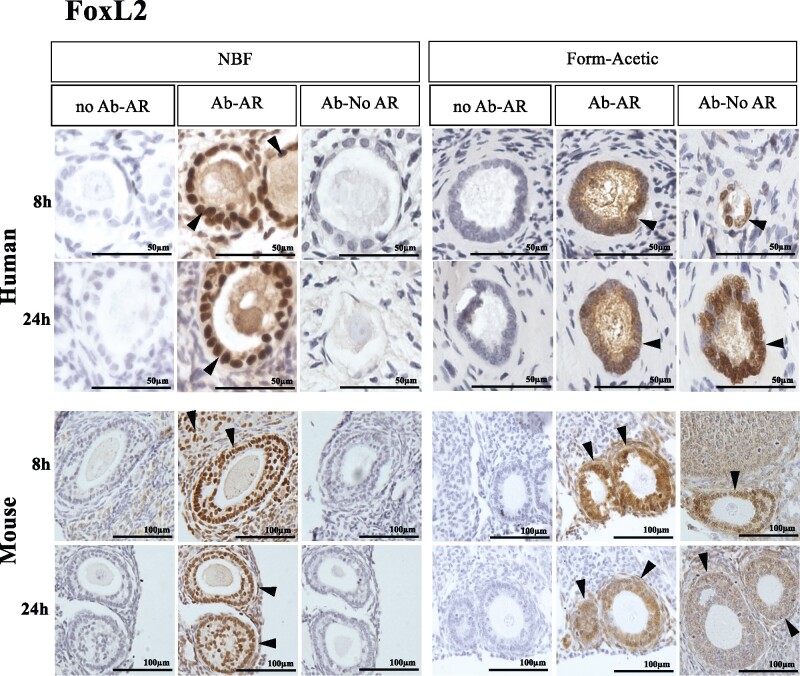
**Detection of FoxL2 using IHC in human and mouse ovarian tissue preserved with different fixatives.** Following the fixation of ovarian tissue in NBF and Form-Acetic (NBF with 5% acetic acid) solutions for 8 h and 24 h, IHC was performed on human and mouse ovarian sections. Fork-head box protein L2 (FoxL2, a transcription factor) was detected in the granulosa cells of follicles. Follicles/cells positively stained are indicated by the arrowheads. No Ab-AR: no antibody was applied on the section treated with AR (control), Ab-AR: antibody was applied, and an AR step was performed, Ab-No AR: antibody was applied, and no AR step was performed. FoxL2 images are representative of experiments using human ovarian cortex samples, n=4 patients per condition (fresh n=2, frozen n=2), and mouse ovaries, n=3 animals per condition. IHC experiments were performed at least twice for each sample. NB: The 12 images in each panel for a single species were all from the same experiment with the same DAB exposure enabling direct comparison between the samples.

FoxL2 was detected in the granulosa cells of transitional to pre-antral stage follicles of NBF and Form-Acetic fixed human and murine samples that had been subject to AR (Fig. 8). Staining was also observed in the ooplasm of follicles in human sections (Fig. 8). Without AR, FoxL2 was only detected in Form-Acetic fixed human and mouse samples but not NBF fixed (Fig. 8).

### Form-Acetic allows detection of extracellular matrix components

**Figure 9.**
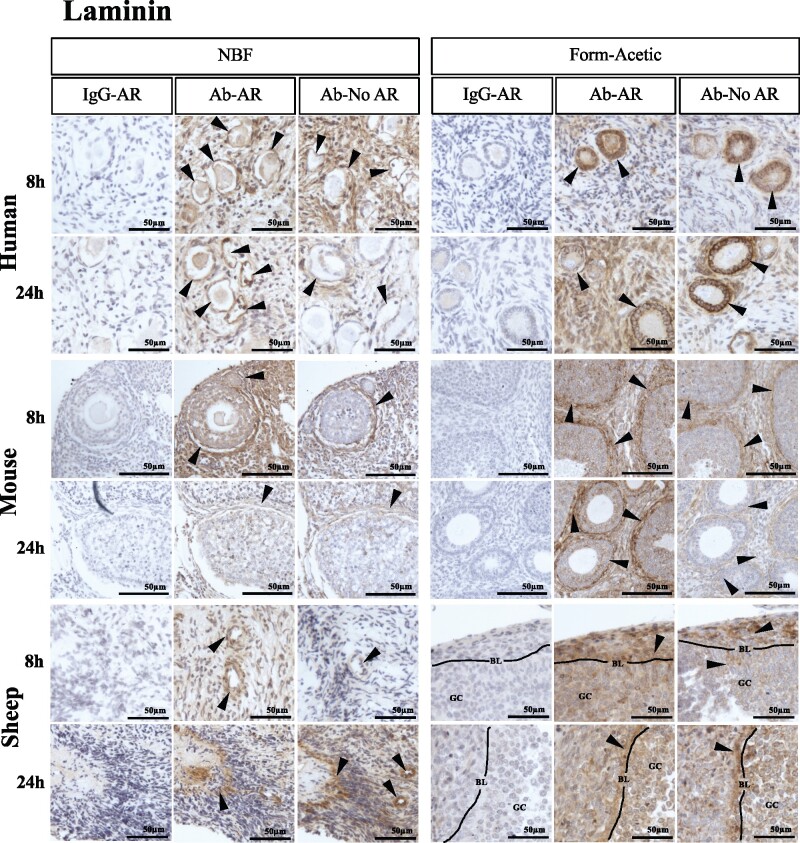
**Detection of laminin using IHC in human, mouse, and sheep ovarian tissue preserved with different fixatives.** Following the fixation of ovarian tissue in NBF and Form-Acetic (NBF with 5% acetic acid) solutions for 8 h and 24 h, IHC was performed on human, mouse and sheep ovarian sections to detect laminin in follicles of all developmental stages, blood vessels and the stroma. Follicles/cells/regions positively stained are indicated by the arrowheads (GC, granulosa cell; BL, basal lamina). IgG-AR: isotype IgG antibody control was applied on the section treated with AR, Ab-AR: antibody was applied, and an AR step was performed, Ab-No AR: antibody was applied, and no AR step was performed. Images are representative of experiments using human ovarian cortex samples, n=3 patients per condition (fresh n=2, frozen n=1), sheep ovarian cortex, n=3 animals per condition, and mouse ovaries, n=3 animals per condition. IHC experiments were performed at least twice for each sample. NB: The 12 images in each panel for a single species were all from the same experiment with the same DAB exposure enabling direct comparison between the samples.

**Figure 10.**
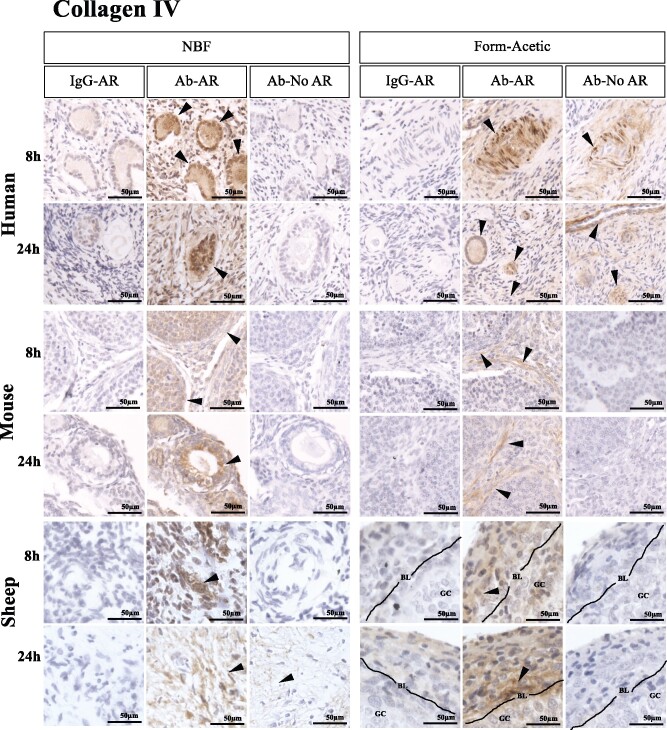
**Detection of Collagen IV using IHC in human, mouse and sheep ovarian tissue preserved with different fixatives.** Following the fixation of ovarian tissue in NBF and Form-Acetic (NBF with 5% acetic acid) solutions for 8 h and 24 h, IHC was performed on human, mouse and sheep ovarian sections to detect collagen IV in follicles of all developmental stages, blood vessels and the stroma. Follicles/cells/regions positively stained are indicated by the arrowheads. IgG-AR: isotype IgG antibody control was applied on the section treated with AR, Ab-AR: antibody was applied, and an AR step was performed, Ab-No AR: antibody was applied, and no AR step was performed. Images are representative of experiments using human ovarian cortex samples, n=3 patients per condition (fresh n=2, frozen n=1), sheep ovarian cortex, n=3 animals per condition, and mouse ovaries, n=3 animals per condition. IHC experiments were performed at least twice for each sample. NB: The 12 images in each panel for a single species were all from the same experiment with the same DAB exposure enabling direct comparison between the samples.

Following AR, both laminin and collagen IV were detected in follicular cells, basement membranes, blood vessels and within the stroma for both NBF and Form-Acetic fixed human, mouse and sheep ovarian sections after 8 and 24 h fixation (Figs 9 and 10). Without AR, laminin was detected in both NBF and Form-Acetic fixed sections from human, mouse and sheep ovaries (Fig. 9). However, without AR, collagen IV was robustly detected only in Form-Acetic fixed human ovarian sections and not NBF fixed human tissue and not in mice or sheep tissue prepared in either fixative (Fig. 10).

### Form-Acetic facilitates detection of a soluble glycoprotein hormone—AMH

**Figure 11.**
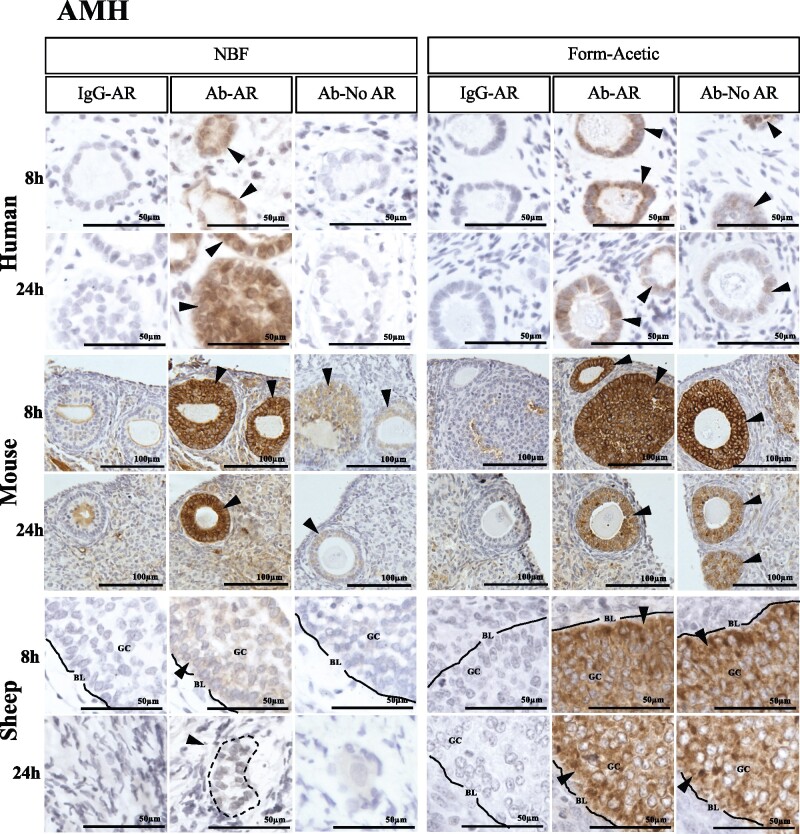
**Detection of anti-Müllerian hormone using IHC in human, mouse and sheep ovarian tissue preserved with different fixatives.** Following the fixation of ovarian tissue in NBF and Form-Acetic (NBF with 5% acetic acid) solutions for 8 h and 24 h, IHC was performed on human, mouse, and sheep ovarian sections to detect anti-Müllerian hormone (AMH) in the granulosa cells of growing follicles. Follicles/cells positively stained are indicated by the arrowheads (GC, granulosa cell; BL, basal lamina). IgG-AR: isotype IgG antibody control was applied on the section treated with AR, Ab-AR: antibody was applied, and an AR step was performed, Ab-No AR: antibody was applied, and no AR step was performed. Images are representative of experiments using human ovarian cortex samples, n=5 patients per condition (fresh n=3, frozen n=2), sheep ovarian cortex, n=3 animals per condition, and mouse ovaries, n=3 animals per condition. IHC experiments were performed at least twice for each sample. NB: The 12 images in each panel for a single species were all from the same experiment with the same DAB exposure enabling direct comparison between the samples with the exception of sheep Form-Acetic 24 h Ab-No AR.

For AMH, a robust signal was observed following AR treatment in and around the granulosa cells of human primary follicles and mouse pre-antral stage follicles after Form-Acetic and NBF fixation (Fig. 11). AMH was also strongly detected after AR in sheep tissues fixed for 8 h in Form-Acetic, but less so for those fixed in NBF; images are from the same experiment. For 24 h sheep samples, a robust signal was observed in granulosa cells of Form-Acetic fixed follicles, however owing to the lack of a comparable follicle in the NBF fixed sheep ovaries, a direct comparison cannot be made between NBF and Form-Acetic fixed tissues. When AR was not performed, AMH was robustly detected in Form-Acetic fixed mouse and sheep sections but more weakly in human sections, whereas after NBF fixation, a weak signal was observed only in mouse tissues and not in human or sheep tissues (Fig. 11).

**Figure 12.**
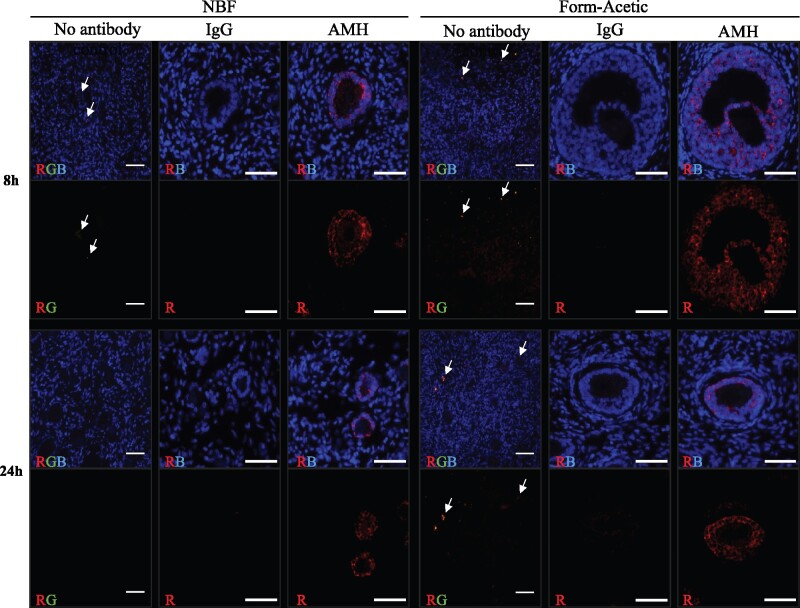
**Detection of AMH using IHC with fluorescent detection in human ovarian tissue preserved with different fixatives.** Following the fixation of human ovarian tissue in NBF and Form-Acetic (NBF with 5% acetic acid) solutions for 8 h and 24 h, IHC using antigen retrieval to detect AMH was performed to detect the glycoprotein in the granulosa cells of follicles. AMH (red) was detected using streptavidin conjugated Alexa Fluor 568 and nuclei (blue) were counterstained with DAPI. Images were processed identically in ImageJ to adjust qualitative aspects (brightness/contrast) to ensure high signal visibility according to best-practice guidelines (see methods section for details). AMH was detected in granulosa cells of growing follicles, while no signal was observed in the negative control (not treated with antibodies) and IgG control (rabbit IgG instead of primary antibody). Autofluorescence caused by lipofuscin (arrows) was visible across the green-red spectrum in oocytes and stroma in both NBF (8 h) and Form-Acetic (8 h and 24 h) fixed tissues (R = red, G = green, B = blue). Images are representative of experiments using human ovarian cortex samples, n=3 (n=2, fresh and n=1, frozen). Each scale bar is 50 μm.

Form-Acetic also proved compatible with IF, as AMH was detected in the granulosa cells of both NBF and Form-Acetic fixed human sections after 8 and 24 h fixation (Fig. 12). Autofluorescence caused by lipofuscin was present in oocytes and stroma in both NBF (8 h) and Form-Acetic (8 and 24 h) fixed tissues but appeared more abundant in Form-Acetic fixed samples (Fig. 12).

### Form-Acetic fixation does not inhibit the TUNEL assay

**Figure 13.**
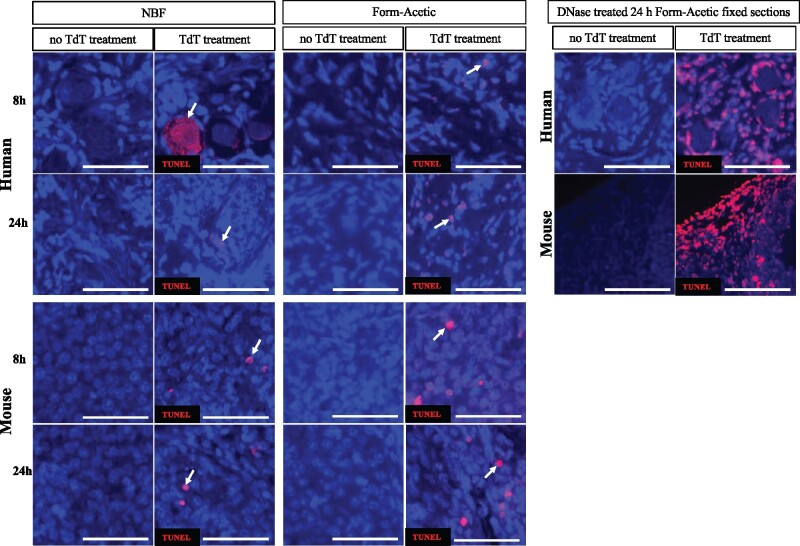
**Representative images of human and mouse ovarian tissue preserved with different fixatives and subjected to the TUNEL assay.** TUNEL was performed on 8 h and 24 h NBF and Form-Acetic (NBF with 5% acetic acid) fixed human and mouse ovarian sections. TUNEL-positive cells (red) were detected using Alexa Fluor 594 and are denoted with white arrows while cellular nuclei (blue) were counterstained with DAPI (TdT, terminal deoxynucleotidyl transferase). Positive control sections were treated with 1 IU/mL DNase I. Images are representative of experiments using human ovarian cortex samples, n=2 (n=1, fresh and n=1, frozen) and for mouse, n=2 per condition. Each scale bar is 100 µm.

To validate the compatibility of Form-Acetic fixation in detecting double-stranded DNA breaks, the TUNEL assay was performed. Fragmented DNA was distinctly labelled after both 8 and 24 h of fixation in both NBF and Form-Acetic mouse and human ovarian tissue (Fig. 13). The TUNEL assay was also performed multiple times using DAB as the means of detecting fragmented DNA and, again, we observed TUNEL-positive cells at both time points for both NBF and Form-Acetic fixed mouse ovarian sections (data not shown).

## Discussion

The study aimed to develop a fixative that not only preserved ovarian tissue morphology without introducing artefact but also could enable downstream histological molecular assays involving protein and nuclear material detection. We identified that fixation with Form-Acetic preserved ovarian tissue morphology and in most cases was capable of reducing artefact in three different species. We also demonstrated that Form-Acetic showed compatibility with multiple histological stains, including H&E, PAS and Masson’s trichrome, allowing for clear detailing of microstructures within fixed sections. Critically, tissues from the three species proved to be suitable for IHC and a wide range of proteins (FOXO3a, FoxL2, collagen IV, laminin, and AMH) were detected in tissue fixed with Form-Acetic, consistent with NBF in this study and studies by other groups using NBF (Yamada *et al.*, 1999; Berkholtz *et al.*, 2006; John *et al.*, 2008; Pisarska *et al.*, 2010; Campbell *et al.*, 2012; Kallio *et al.*, 2012; Heeren *et al.*, 2015; Saatcioglu *et al.*, 2016; Fujisawa *et al.*, 2018; Eivazkhani *et al.*, 2019; Ouni *et al.*, 2019). It was also noted that proteins were detected similarly in both frozen-thawed and fresh human tissue fixed in Form-Acetic. Lastly, we established that AMH detection was equivalent in Form-Acetic fixed sheep and human ovarian sections prepared 14 months earlier compared to those freshly prepared. Based on our results, we, therefore, recommend the use of Form-Acetic as a fixative in ovarian research.

Shrinkage introduced by NBF poses a concern, occurring in diverse tissue types ranging from 50% in oesophageal tissue (Siu *et al.*, 1986) to 34% in breast tissue (Yeap *et al.*, 2007). In this study, we did not measure loss in tissue volume following NBF fixation but compared artefact levels in NBF fixed tissue to Form-Acetic fixed tissue, which presented minimal artefact. It has been proposed by Jones (1972) that the shrinkage seen in NBF-fixed tissue, especially breast and fatty tissue, may be a consequence of lipid degradation by formaldehyde into its water-soluble components and explains that subsequent cellular dehydration presents the shrinkage that we observe during histology. With Form-Acetic, however, this shrinkage does not occur owing to the properties of acetic acid. Acetic acid is a frequent component of fixative solutions, including Bouin’s, and is known to counteract the shrinkage introduced by picric acid in Bouin’s solution (Howat and Wilson, 2014). It is likely that this enriching property of the acid is responsible for the reduced proportion of artefact in Form-Acetic fixed tissue as observed in this study. It is also possible that the acetic acid might be protecting the tissue during the post-fixation processing events that can also cause shrinkage (Baker, 1958).

Acetic acid is a weak acid and its presence in Form-Acetic rendered AR redundant for some IHC analyses, potentially related to the ability of acetic acid to break down cross-linkages between protein molecules (Baker, 1958). Borkar *et al.* (2011) detailed the denaturing property of 9 M glacial acetic acid in the presence of the HIV protease, showing a direct interaction between certain regions of the protein and acetic acid molecules. In our study, 0.874 M acetic acid was used; therefore, it is possible that the concentration and quick penetration rate of acetic acid within the fixative is sufficient to preserve epitopes that would otherwise be altered by formaldehyde fixation but not denature the protein thereby enabling protein detection without AR.

Acetic acid cross-links nucleoproteins but not cytoplasmic proteins and is also known not to preserve the Golgi apparatus or mitochondria (Baker, 1958); presumably equivalently in Bouin’s and Form-Acetic. It is thereby important to conduct further studies to observe and detail the interaction of the fixative with these cellular structures using Form-Acetic with different acetic acid concentrations. Interestingly, Form-Acetic appeared to better preserve lysosomes as lipofuscin autofluorescence was more abundant in oocytes and the stroma of the Form-Acetic fixed tissues. To determine if the lipofuscin signal could be removed to enable other molecules in the oocyte to be investigated using fluorescence, we used commercially available quenching products and successfully removed the autofluorescence signal of lipofuscin (data not shown).

As previously stated, the ideal fixative should enable DNA/RNA preservation for sequencing. NBF directly interacts with nuclear material and results in poor detection (Bresters *et al.*, 1994), sequence alterations (Williams *et al.*, 1999), and even degradation (Srinivasan *et al.*, 2002) of the genetic content. The molecular interaction that Form-Acetic may have with nuclear material is not completely known as we only examined whether fixation with Form-Acetic enabled the detection of fragmented DNA. However, Baker and Silverton (1976) reported on the ability of acetic acid to precipitate nuclear-material and identified it as a useful tool for studying DNA/RNA content. It is thereby probable that Form-Acetic may preserve nuclear material composition and organisation for molecular studies including DNA/RNA extraction, amplification, and sequencing to generate quality sequences or detection.

In addition to the primary objectives, we also observed that Form-Acetic benefited the tissue preparation and the embedding process. Hardening of NBF fixed tissues, particularly for the human and sheep samples, meant that these samples sometimes ‘fell out’ of the paraffin wax during sectioning, leaving empty areas in the wax ribbons. When a tissue was behaving like this, blocks containing fixed samples had to be hydrated in ice water in-between sectioning to counteract this event. Sectioning difficulty such as this was not encountered with Form-Acetic and Bouin’s fixed samples, because of the properties of acetic acid which prevent hardening of fixed tissues caused by alcohol treatment during sample processing (Baker, 1958).

Based on our observations and the data presented here, we propose that Form-Acetic becomes the fixative of choice for histo-morphological analyses. From the results, we recommend fixation of whole mouse ovaries, or ovarian tissue pieces from large mammals (approximately 1 mm^3^) for 8–24 h for both routine histology and histological molecular analysis. However, 24 h fixation in Form-Acetic is a highly convenient duration for subsequent collection and does not inhibit antigen availability. Fixation times and volumes may need to be optimised for larger tissue pieces or different tissue types. Penetration of acetic acid is rapid and, therefore, diffusion of NBF is likely to be the limiting factor when estimating fixation time. Finally, as is currently the case for NBF, determining IHC protocols for different antigens will likely require optimisation.

In summary, we have defined the use of a fixative known as Form-Acetic, that is able to preserve ovarian tissue morphology in a manner superior to the most commonly used fixatives for multiple species. The fixative is simple to prepare, requiring only two commonly available reagents; NBF and acetic acid. We have demonstrated that the fixative is compatible with common histological staining methods in human, mouse and sheep tissues. Critically, the fixative is also compatible with the detection of a range of proteins using IHC and, for some proteins, AR steps are unnecessary unlike NBF. In addition, we have confirmed that the fixative allows for detection of fragmented DNA using the TUNEL assay. We, therefore, recommend that Form-Acetic replaces currently used fixatives and encourage others to introduce it into their research workflows.

## Data availability

The data underlying this research article can be made available upon reasonable request to the corresponding author.

## Supplementary Material

deab075_Supplementary_Table_S1

deab075_Supplementary_Table_S2

deab075_Supplementary_Table_S3

deab075_Supplementary_Figure_S1
