# Supplementary material for: Improved preservation of ovarian tissue morphology that is compatible with antigen detection using a fixative mixture of formalin and acetic acid
Source: Hum Reprod. 2021 May 6;36(7):1871–90. doi: 10.1093/humrep/deab075 (PMC8213453; doi:10.1093/humrep/deab075)
Supplement: deab075_Supplementary_Table_S1 [file deab075_supplementary_table_s1.pdf]

Human Reproduction, pp. 1–1, 2021
doi:10.1093/humrep/deab075

human reproduction

SUPPLEMENTARY DATA

Supplementary Table SI **Results of statistical comparison for integrity of tissue after different fixation conditions in human ovarian tissue.**

Conditions		N of sections/follicles	NBF			Bouin's			Form-Acetic		
			4 h	8 h	24 h	4 h	8 h	24 h	4 h	8 h	24 h
			**Human—Follicle Integrity**								
NBF	4 h	35	–								
	8 h	45	*ns*	–							
	24 h	59	*ns*	*ns*	–						
Bouin's	4 h	29	***	*ns*	*ns*	–					
	8 h	35	*ns*	***	*ns*	*ns*	–				
	24 h	19	*ns*	*ns*	*	*ns*	*ns*	–			
Form-Acetic	4 h	8	***	*ns*	*ns*	*ns*	*ns*	*ns*	–		
	8 h	99	*ns*	***	*ns*	*ns*	*ns*	*	*ns*	–	
	24 h	123	*ns*	*ns*	***	*ns*	*	**	*ns*	*ns*	–
			**Human—Follicle-Stroma Integrity**								
NBF	4 h	35	–								
	8 h	45	*ns*	–							
	24 h	59	*ns*	*ns*	–						
Bouin's	4 h	29	***	*	*ns*	–					
	8 h	35	*	***	*	*ns*	–				
	24 h	19	*ns*	*	***	*ns*	*ns*	–			
Form-Acetic	4 h	8	***	*ns*	*ns*	*	*	*ns*	–		
	8 h	99	*ns*	***	*ns*	*	*ns*	*ns*	*ns*	–	
	24 h	123	*ns*	*ns*	***	*ns*	*ns*	**	*ns*	*ns*	–
			**Human—Stroma Integrity**								
NBF	4 h	26	–								
	8 h	26	**	–							
	24 h	34	***	***	–						
Bouin's	4 h	26	***	***	***	–					
	8 h	35	***	***	*ns*	**	–				
	24 h	22	***	*ns*	***	**	***	–			
Form-Acetic	4 h	20	***	*ns*	***	***	*	*ns*	–		
	8 h	49	*ns*	***	***	*	*ns*	***	*ns*	–	
	24 h	55	***	***	***	*ns*	***	***	*ns*	*ns*	–

'ns' indicates no significance;.
*$P < 0.05$; **$P < 0.005$; ***$P < 0.001$; ****$P < 0.0001$.
NBF: neutral buffered formalin.
follicle integrity: the amount of clear space, as a result of cellular shrinkage due to fixation, observed within the follicle.
follicle-stroma integrity: space between the follicle and the surrounding stroma.
stroma integrity: space between stromal cells.
