# Supplementary material for: Improved preservation of ovarian tissue morphology that is compatible with antigen detection using a fixative mixture of formalin and acetic acid
Source: Hum Reprod. 2021 May 6;36(7):1871–90. doi: 10.1093/humrep/deab075 (PMC8213453; doi:10.1093/humrep/deab075)
Supplement: deab075_Supplementary_Table_S2 [file deab075_supplementary_table_s2.pdf]

Human Reproduction, pp. 1–1, 2021
doi:10.1093/humrep/deab075

human reproduction

SUPPLEMENTARY DATA

Supplementary Table SII **Results of statistical comparison for integrity of tissue after different fixation conditions in mouse ovarian tissue.**

Conditions		N of sections/follicles	NBF			Bouin's			Form-Acetic		
			4 h	8 h	24 h	4 h	8 h	24 h	4 h	8 h	24 h
**Mouse—Follicle Integrity**											
NBF	4 h	28	–								
	8 h	34	*ns*	–							
	24 h	37	*ns*	*ns*	–						
Bouin's	4 h	34	***	*ns*	*ns*	–					
	8 h	44	*ns*	***	*ns*	*ns*	–				
	24 h	44	*ns*	*ns*	***	*ns*	*ns*	–			
Form-Acetic	4 h	36	***	*ns*	*ns*	*ns*	*ns*	*ns*	–		
	8 h	43	*ns*	***	*ns*	*ns*	*ns*	*ns*	*ns*	–	
	24 h	57	*ns*	*ns*	***	*ns*	*ns*	*ns*	*ns*	*ns*	–
**Mouse—Follicle-Stroma Integrity**											
NBF	4 h	28	–								
	8 h	34	*ns*	–							
	24 h	37	*ns*	*ns*	–						
Bouin's	4 h	34	***	*ns*	*ns*	–					
	8 h	44	*ns*	***	*ns*	*ns*	–				
	24 h	44	*ns*	*ns*	***	*ns*	*ns*	–			
Form-Acetic	4 h	36	***	*ns*	*	*ns*	*ns*	*	–		
	8 h	43	*ns*	***	*ns*	*ns*	*ns*	*ns*	*ns*	–	
	24 h	57	*	*ns*	***	*	*ns*	*ns*	*ns*	*ns*	–
**Mouse—Stroma Integrity**											
NBF	4 h	12	–								
	8 h	10	**	–							
	24 h	11	*ns*	*ns*	–						
Bouin's	4 h	10	***	*ns*	*ns*	–					
	8 h	10	*ns*	***	*ns*	*ns*	–				
	24 h	10	*ns*	*ns*	***	*ns*	*ns*	–			
Form-Acetic	4 h	9	***	*	*ns*	*ns*	*ns*	*ns*	–		
	8 h	11	*	***	*ns*	*ns*	*ns*	*ns*	*ns*	–	
	24 h	11	*ns*	*ns*	***	*ns*	*ns*	*ns*	*ns*	*ns*	–

'ns' indicates no significance;.
*$P < 0.05$; **$P < 0.005$; ***$P < 0.001$; ****$P < 0.0001$.
