# Supplementary material for: Improved preservation of ovarian tissue morphology that is compatible with antigen detection using a fixative mixture of formalin and acetic acid
Source: Hum Reprod. 2021 May 6;36(7):1871–90. doi: 10.1093/humrep/deab075 (PMC8213453; doi:10.1093/humrep/deab075)
Supplement: deab075_Supplementary_Figure_S1 [file deab075_supplementary_figure_s1.pdf]

Human Reproduction, pp. 1–1, 2021
doi:10.1093/humrep/deab075

human reproduction

SUPPLEMENTARY DATA

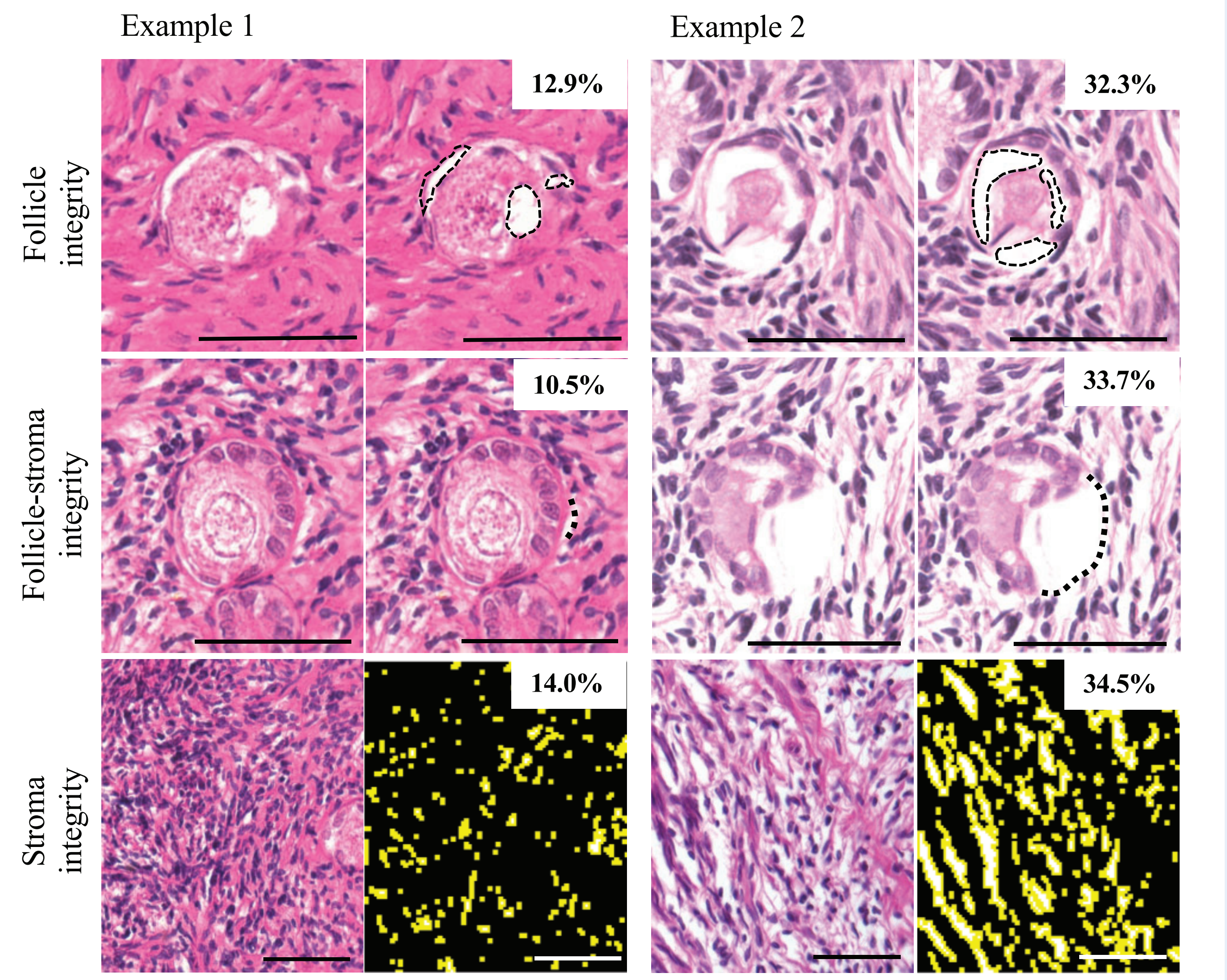

Supplementary Figure S1. Method for histological assessment of ovarian tissue prepared with different fixatives. Three histological assessments were performed which involved determining the integrity of the follicle (FI), follicle-stroma (FSI) and stroma (SI). The degree of artefact (described as clear space) was measured on ImageJ. Shown here are the assessed categories and representative images displaying various degrees of artefact. Composite images detail the method in which the subject of that category was assessed and numbers within figures correspond to the proportion of artefact measured. For FI, the total area of artefact (dotted shapes) was measured as a percentage of the total follicle area. For FSI, the perimeter of non-interaction between the follicle and stroma (dotted line) was calculated as a percentage of the total perimeter of the follicle while SI was determined by calculating the total area of artefact as a percentage of the total stroma for a section, using thresholding. Thresholding involved the conversion of images to 8 bits, which changed the coloured image to black and white and threshold values were adjusted using the original colour image as a reference to discriminate ‘clear spaces’ (artefact) from darkly stained regions. White regions, representing artefact were highlighted (in yellow) and measured. Each scale bar is 50 µm.
